# C-853-04. Early Mobilization After Split-Thickness Skin Grafting Is Not Associated with Graft Loss in Burn Patients

**DOI:** 10.1093/jbcr/irag033.072

**Published:** 2026-04-06

**Authors:** Maybelle E Singson, Desiree Pinto, Kate F Wallace, Emily G Woolhiser, Katelyn Marzo, Tuan D Le, Bonnie C Carney, Lauren T Moffatt, Jamie G Oh, Taryn E Travis, Jeffrey W Shupp, Shawn Tejiram

**Affiliations:** MedStar Washington Hospital Center, Washington, DC; MedStar Georgetown University Hospital / Washington Hospital Center, Washington, DC; MedStar Washington Hospital Center, Washington, DC; Memorial Hospital System, Davie, FL; Marymount University, Dumfries, VA; The Burn Center at MedStar Washington Hospital Center, Washington, DC; The Burn Center at MedStar Washington Hospital Center, Washington, DC; The Burn Center at MedStar Washington Hospital Center, Washington, DC; The Burn Center at MedStar Washington Hospital Center, Washington, DC; The Burn Center at MedStar Washington Hospital Center, Washington, DC; MedStar Washington Hospital Center, Washington, DC; MedStar Washington Hospital Center, Washington, DC

## Abstract

**Introduction:**

Split thickness skin grafting (STSG) remains standard of care for wound closure of deep and partial thickness wounds. Immobilization after STSG was historically utilized for graft loss prevention, with a majority of recently surveyed burn surgeons reporting immobilization practices until post-operative day (POD) 3 or later. Previous literature has explored feasibility of mobility earlier than POD3 following STSG without increased rates of graft loss. However, these studies are limited to small graft sizes, weightbearing limitations, lower extremity STSG, and non-critically ill patients. The aim of this study was to evaluate the association between time to mobilization after STSG and graft loss. It was hypothesized that early mobilization (POD1 and earlier) would not be associated with increased risk of graft loss.

**Methods:**

Patients who underwent STSG for treatment of thermal burn injuries at an adult ABA-verified burn center between August 2021 to August 2024 were retrospectively reviewed. Each incidence of STSG was individually categorized by day of mobilization as early (POD0-1) or late (POD2 or later). STSG mobilization cohorts were then compared to identify rates of major graft loss using an institutional grading scale, defined as graft loss >50% of area grafted, graft loss requiring prolonged wound care (>5 days), or graft loss requiring re-operation.

**Results:**

A total of 240 patients met inclusion criteria, with a total of 431 incidences of STSG. Of these, 392 were assigned to the early mobilization group and 39 to the late mobilization group. There was no difference between groups in terms of demographics, injury characteristics or surgical interventions, except for time to autograft. Median time to burn excision was 3 days in both groups. Nearly half of the grafts (48.7%) crossed a joint. Negative pressure wound therapy was used as a post-op dressing in most patients (92.3%). Among STSG incidences, 193 occurred in critically ill patients (44.7%). Rates of graft loss were similar between groups (9% vs 9.2%, *p*=.93). Early mobilization did not increase the odds of graft loss (OR 0.97; 95% CI, 0.47-2.01; *p*=.93). Ambulation, compared to in-bed exercises, was not associated with increased odds of graft loss (OR 1.63; 95% CI, 0.48-5.58; *p*=.44). The highest level of mobility achieved was not significantly different between groups. Graft location (lower vs. upper extremity) was not associated with increased odds of graft loss (OR 1.17; 95% CI, 0.53-2.60; *p*=.69).

**Conclusions:**

The findings suggest that early mobilization within 24 hours after STSG does not increase the risk of graft loss in burn patients and out of bed ambulation does not increase the risk of graft loss compared to in-bed therapy alone. Further prospective studies are needed to better elucidate the relationship.

**Applicability of Research to Practice:**

Understanding graft loss risk in tandem with patient rehabilitation needs can assist burn teams with rehabilitation planning and timing following STSG.

**Funding for the study:**

N/A.

